# Internal wave turbulence at a biologically rich Mid-Atlantic seamount

**DOI:** 10.1371/journal.pone.0189720

**Published:** 2017-12-21

**Authors:** Hans van Haren, Ulrike Hanz, Henko de Stigter, Furu Mienis, Gerard Duineveld

**Affiliations:** Royal Netherlands Institute for Sea Research (NIOZ) and Utrecht University, Den Burg, the Netherlands; CNRS, FRANCE

## Abstract

The turbulence regime near the crest of a biologically rich seamount of the Mid-Atlantic Ridge southwest of the Azores was registered in high spatial and temporal resolution. Internal tides and their higher harmonics dominate the internal wave motions, producing considerable shear-induced turbulent mixing in layers of 10–50 m thickness. This interior mixing of about 100 times open-ocean interior values is observed both at a high-resolution temperature sensor mooring-site at the crest, 770 m water depth being nearly 400 m below the top of the seamount, and a CTD-yoyo site at the slope off the crest 400 m horizontally away, 880 m water depth. Only at the mooring site, additionally two times higher turbulence is observed near the bottom, associated with highly non-linear wave breaking. The highest abundance of epifauna, notably sponges, are observed just below the crest and 100 m down the eastern slope (700–800 m) in a cross-ridge video-camera transect. This sponge belt is located in a water layer of depressed oxygen levels (saturation 63±2%) with a local minimum centered around 700 m. Turbulent mixing supplies oxygen to this region from above and below and is expected to mix nutrients away from this biodegraded layer towards the depth of highest abundance of macrofauna.

## Introduction

Seamounts display various physical oceanographic processes, which have an effect on biogeochemical processes resulting in a large variability in the abundance of life [1]. While some are hotspots teeming with life such as sponges, cold-water corals and fish on particular slopes and on their summits, others are barren of life [2]. Environmental conditions such as geomorphology, geological origin and age, local hydrodynamic regime, light levels, water chemistry, food supply and primary productivity in the overlying water determine the discrimination between potentially suitable seamount habitats for, for instance, cold-water corals or sponges [3–5]. The variations in these environmental conditions can also result in distinct vertical distributional patterns of fauna, like coral and sponges, across the seamount, e.g. the Condor seamount near the Azores NE Atlantic Ocean [4,6,7].Environmental properties favoring benthic fauna like temperatures are found to be within the range of [5, 14]°C near summits of seamounts, which corresponds to water depths between about 500 and 1,500 m at mid-latitudes, and a few 100 m shallower in tropical regions e.g., [8]. More importantly, but also less known, are the conditions leading to sufficient food and oxygen supply, which are vital for deep-sea ecosystems. Leading physical processes seem to be internal wave motions supported by the density stratification that is still moderate-strong at these depths. In particular, over tops of confined, small seamounts trapped internal hydraulic jumps can provide strong turbulent overturning [9]. Following 2D idealized modelling, such mixing leads oxygen supply over tops of cold-water coral mounds, provided the mounds are a few 100 m high with slopes that are steeper than the leading internal wave slope [5].

Here, we aim to learn more on the interaction between a steep confined seamount (sensu [10]), located in the Mid-Atlantic Ridge (southwest of the Azores), internal wave mixing and its relation to the presence of macro fauna. To this extent, a 200 m high temperature sensor array was moored on the crest of an elongated seamount. Near the mooring a 13 h CTD-yoyo station was employed to measure the variability of other environmental parameters including oxygen. Between the two sites, perpendicular to the elongated seamount and crossing the crest, a tethered video-camera transect was made to identify the abundance of the benthic fauna on the slopes of the seamount.

## Data

**Fig 1:**
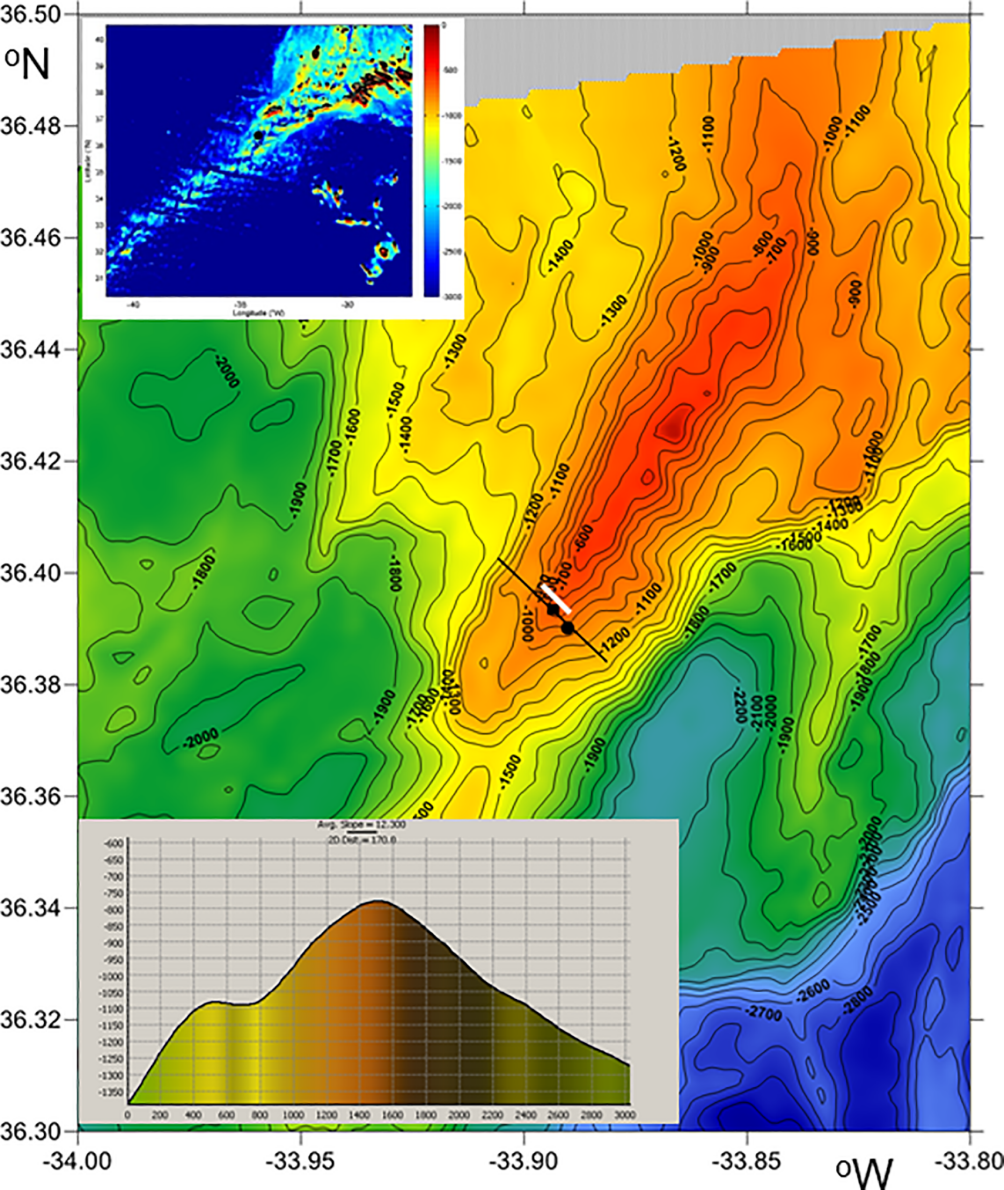
Small seamount of the Mid-Atlantic Ridge, northwest of the Azores islands. Bathymetry from R/V Pelagia’s multibeam system. Solid black contours every 100 m. The black line indicates the cross-section (lower insert) through the black diamond mooring location and the black dot CTD-yoyo position. The video transect was more or less parallel to this line, slightly to the northeast (white line). Insert: larger area bathymetry using the 9.1 ETOPO-1 version of[43], with black dot indicating the site of operations. Colours indicate different depth ranges.

Ten days of high-resolution moored observations have been made near the crest of an elongated seamount bounding the axial graben of the Mid-Atlantic Ridge 400 km southwest of the Azores (Fig 1). At its shallowest point the seamount is 395 m high, towering about 3 km above the bottom of the axial graben. A 230 m high mooring was deployed at 36° 23.56′ N, 33° 53.62′ W in 770 m water depth on 30 June 2016 and recovered on 10 July, 2016, during RV Pelagia cruise 64PE412 carried out in the framework of the TREASURE project. Longer mooring duration was not feasible because of fisheries risk, but a suitable number of tidal periods was covered for some statistics. This position is at the crest, some 5 km southwest of and 350 m deeper than the top of the seamount. The original intention was to deploy the mooring on top of the seamount, but this turned out to be impossible because of the abundance of lost long-line fishing-lines on the summit and consequent risk of entanglement of the mooring. Typical bottom slopes β as determined from multibeam echosounder data over a 100 m grid resolution are between 1 and 45°.

Below the single buoyancy package on top providing 240 kg net buoyancy, a 196 m cable holds 98 high-resolution temperature T sensors. The lowest sensor was mounted at 5 m above the bottom (mab). Two Nortek AquaDopp acoustic current meters were 2.5 m above the upper temperature sensor at 201 mab and, clamped to the cable, between the lowest and second-lowest T-sensor at 6 mab. As verified with pressure and tilt sensors, the top of the mooring did not move more than 0.3 m vertically and it never deflected more than 10 m horizontally, under up to 0.5 m s^-1^ current speeds.

High-resolution NIOZ4 T-sensors are used, for characteristics see [11]. The sensors were spaced at 2.0 m intervals and sampled at a rate of 1 Hz. They are synchronized via induction every 4 hours, so that timing mismatch is <0.02 s. Their precision is about 10^−4^°C. Three sensors showed calibration problems or too high noise levels and are not further considered. Their data are linearly interpolated.

The sensors were calibrated at NIOZ using a thermostatic bath with constant temperature levels to within ±10^−4^°C of their preset values. The standard drift correction is requiring a statically stable density (temperature) profile for a section of data of at least the inertial period, in this area 0.84 days, but preferably 2–7 days. The calibrated and drift-corrected data are transferred to Conservative (~potential) Temperature (Θ) values using the GSW-software described in [12].

**Fig 2:**
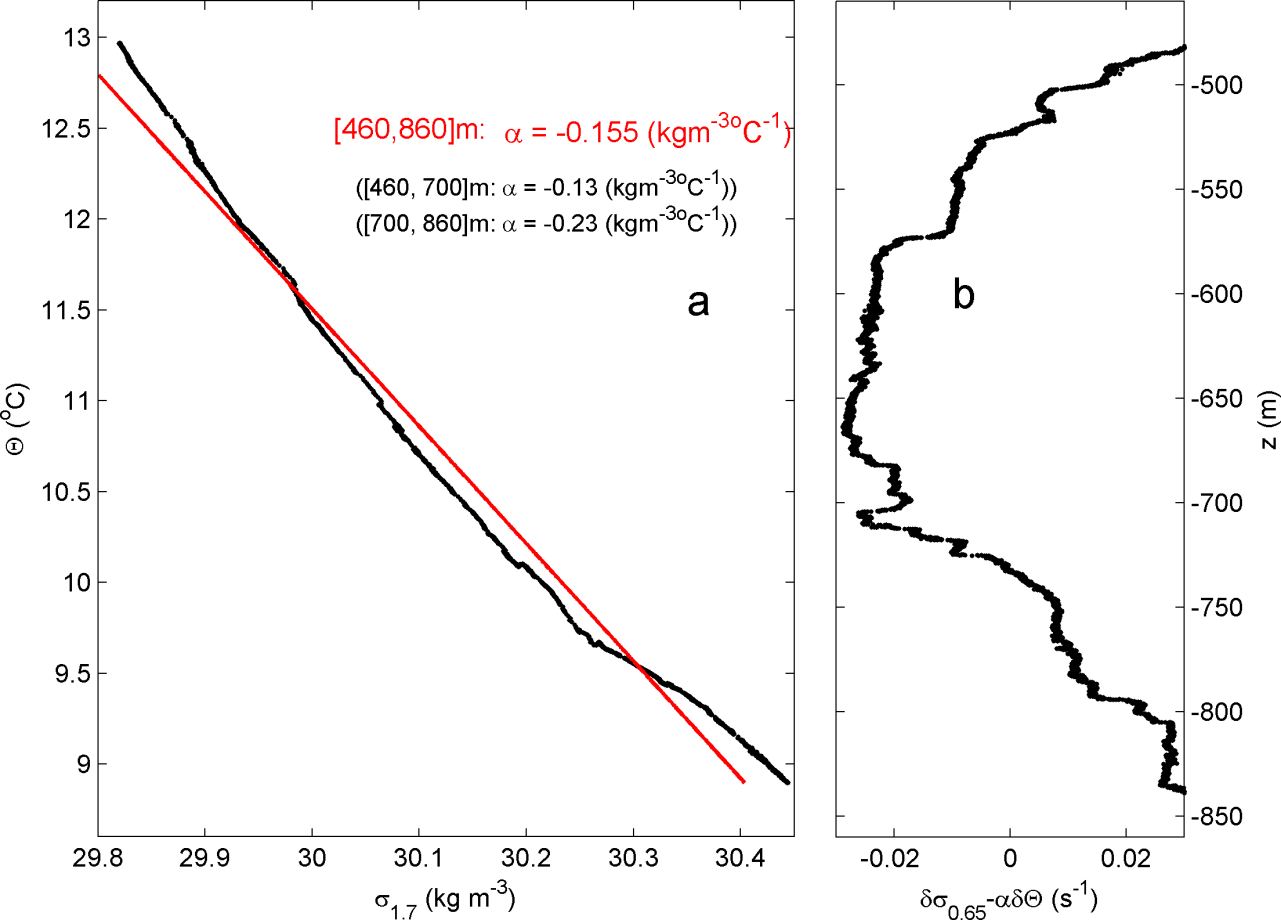
Conservative temperature-density anomaly relationship, referenced to 650 m from CTD-data obtained at 400 m from the mooring on day 186.5. (a) Potential density anomaly versus Conservative Temperature between 460 and 860 m, with two shorter ranges slopes. (b) Comparison of the slope of relationships in a. with density from profile data.

For comparison of the high-resolution moored temperature data with other parameters, shipborne SeaBird-911 Conductivity-Temperature-Depth ‘CTD’-profiles were obtained, sampled at a rate of 24 Hz. Between 05 July 11 UTC and 06 July 00:15 UTC, a total of 33 yoyo-casts were made within 400±40 m SE from the mooring at 36° 23.41′ N, 33° 53.42′ W in 880±20 m water depth. The water depth variation does not reflect the tidal surface height variation of about 1 m crest-trough, but horizontal displacement of the ship over steep bottom slope while performing the CTD casts. The CTD-data are also used to establish the local density-temperature relationship for use of the moored T-sensor data as tracer for potential density anomaly variations, referenced to 650 m, δσ_0.65_ (Fig 2). The relationship was reasonably tight, δσ_0.65_ = αδΘ, α = -0.15±0.3 kg m^-3^°C^-1^. The amount of good T-sensors and the 2-m-resolution were sufficient to use these moored data to estimate turbulence parameters through the resolution of scales of up to the largest energy-containing Ozmidov-scales of 10–50 m from turbulence in stratified fluids.

Turbulence dissipation rate ε = c_1_^2^d^2^N^3^ and vertical eddy diffusivity K_z_ = m_1_c_1_^2^d^2^N were estimated from the T-sensor data using the method of reordering potentially unstable vertical density profiles in statically stable ones, as proposed by [13]. Here, d denotes the displacements between unordered (measured) and reordered profiles. N denotes the buoyancy frequency computed from the reordered profiles. In the highly turbulent, stratified, restratifying and relatively strong shear environment of the present observations, we used standard constant values of c_1_ = 0.8 for the Ozmidov/overturn scale factor and m_1_ = 0.2 for the mixing efficiency [14–16]. See [17] for details on applying the overturning-scale method for estimating turbulence from moored high-resolution T-sensors. For the use with density anomaly data from the CTD-observations, we only used those data of which the absolute value of difference with the local reordered value exceeded a threshold of 3×10^−4^ kg m^-3^, which corresponds to applying a threshold of 1.4×10^−3^ kg m^-3^ to raw data variations e.g., [18]. Hereafter, averaging over the 194 m depth range of moored T-sensors is indicated by *<*… *>*, over time by […].

Video images of the seafloor were recorded with a tethered high-definition HD digital video camera mounted in a sturdy frame, along a northwest-southeast transect from the CTD-yoyo position to the mooring, passing the mooring slightly to the northeast, and across the ridge. The camera was directed vertically to the seafloor and its signal was transmitted via a fibre-optical cable to the ship. In order to quantify the surveyed area two Oktopus™ laser pointers were mounted on the frame for scale. The camera was towed at a speed of approximately 0.15 m s^-1^, whilst being held between 0.5 and 3 m above the seabed. Variations in height are mainly due to surface wave induced ship motions. The video camera additionally saved a picture every 5 s. Pictures were analyzed in tracks of 6 minutes (~72 pictures). A single picture covers a relative horizontal area of roughly 2 x 2 m, depending on slope and height of camera. Therefore, a track of 6 minutes equals an area of about 100 m^2^. Substrate and organisms with a size larger than 0.1 m were noted, including mobile fauna like fish. Due to (quality) restrictions of the video the organisms were logged as morphospecies. A morphospecies group contains organisms that all conform to defined morphological characteristics.

## Observations

**Fig 3:**
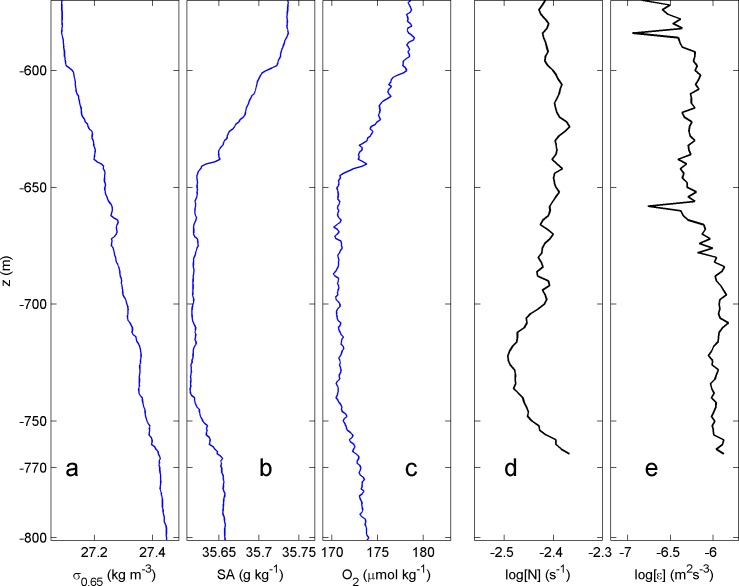
**Typical profiles of several parameters measured at CTD-station (a-c) and the mooring (d-e) for the range of 500–800 m**. (a) Density anomaly referenced to 650 dbar, CTD-profile number 12. (b) Corresponding absolute salinity. (c) Corresponding oxygen levels. (d) Ten day mean buoyancy frequency. (e) Ten day mean turbulence dissipation rate.

A single CTD-profile from the yoyo station shows the water characteristics of the area for the vertical range of moored T-sensors (Fig 3). In density, small-scale instabilities are observed (Fig 3A). The salinity profile (Fig 3B) and the oxygen profile show a local minimum around 700 m. The stratification is more or less constant with depth, both at the CTD-yoyo and at the mooring station (Fig 3D shows the ten-day mean value). The associated ten-day mean turbulence dissipation rate is high, and almost constant over the depth-range of moored temperature sensors (Fig 3E).

### Comparison of yoyo CTD-data with moored T-sensor data

The CTD-package was yoyo-ed between 300 m and about 10 m from the bottom, which varied a few tens of meters reflecting the considerable steepness of slope. One down- and upcast of yoyo took about 24 minutes. The relatively small horizontal distance of the CTD-site from the mooring site, on average 400 m, allows a comparison with the moored T-sensor data, even though the CTD-site is about 100 m deeper.

**Fig 4:**
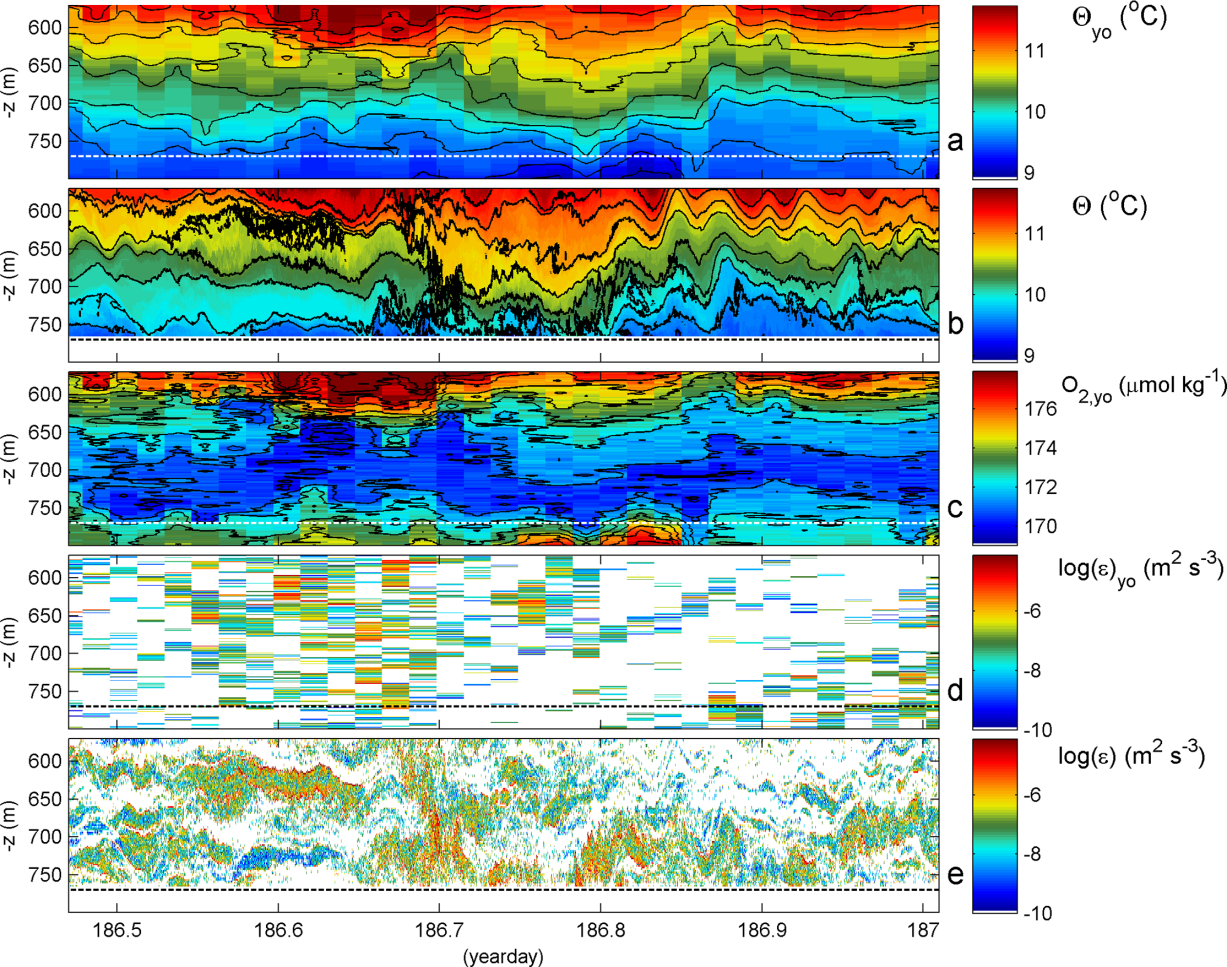
Comparison of 13 hour CTD-yoyo data with moored temperature sensor data obtained 400 m to the northwest, up the slope. (a) Conservative Temperature from CTD-yoyo, with contours every 0.25°C in black. The horizontal white dashed line indicates the bottom for the mooring site, for reference. (b) Conservative Temperature from moored T-sensors, with identical colour range and contour interval as in (a). Local bottom is indicated by a black dashed line. (c) Oxygen content from CTD-yoyo. Black contour lines every 1 μmol kg^-1^. (d) Logarithm of turbulence dissipation rate, inferred from data in (a). (e) Logarithm of turbulence dissipation rate, inferred from data in (b).

In Fig 4A and 4B the two are compared for the parameter Conservative Temperature. The CTD data are binned to 1 m vertical intervals, half the T-sensor interval. The comparison between the two data sets suffers from the much ‘slower’ sampling of the CTD. This is not due to the actual 24-Hz sampling rate of the CTD, but entirely due to the lowering and hoisting of the CTD-package: the CTD moves slantwise through the water at a speed of about 1 m s^-1^, whereas the T-sensor array of 200 m is sampled within 0.02 s, every 1 s. Nevertheless, it is seen that the two show roughly the same temperature changes with depth, which move up and down every 1 and 4 hours, approximately. Small-period variations are observed more or less in phase over the 400 m horizontal distance, e.g., around days 186.5 and 186.85.

Although the two are alike, the yoyo station seems to trail somewhat behind the mooring, during the warming phase until day 186.78. During this phase, ‘near-bottom’ waters are about 0.05°C cooler at the CTD-site. Here, near-bottom is referred as the bottom at the mooring site. This suggests cooler water is lifted at some distance away from topography and pushed down nearer to it. The major dip centered around day 186.78, indicating the turn of tide from warming to cooling phase. It dips considerably deeper near the topography, i.e. at the mooring site. The coarse sampling in time of the yoyo, however, does not allow establishment of a phase difference between arrival of the cool water in a near-bottom front.

The yoyo-data already demonstrate distinct variations with time and depth between the various profiles (Fig 4A), such short-term ‘rugged’ variability is just augmented in the mooring data in Fig 4B. This is especially visible in the temperature contour lines, which are far from smooth for certain periods, at all depth levels. It is indirectly reflected in the oxygen data (Fig 4C), and, naturally, in the dissipation rates (Fig 4D and 4E). An oxygen minimum layer is apparent between 650 and 750 m, with dissolved oxygen content of 170–180 μmol kg^-1^, 61–65% saturation. It coincides with a relative salinity minimum (Fig 3) and a relative nutrient maximum, which is likely due to biodegradation (see for example observations in [8,19]). Towards shallower depths, oxygen increases rapidly, with values up to 200 μmol kg^-1^ (85% saturation at 490 m and up to 230 μmol kg^-1^ (100% saturation) at 100 m. In Fig 4C, oxygen contours seem to follow temperature contours, but there is also evidence of mixing pockets in oxygen, e.g. day 186.58 between 600 and 650 m and the wiggling lining bordering the green of the large depression centered at day 186.78. Around that time and in contrast with temperature, oxygen shows larger values coming from below. It is expected that the higher oxygen levels above and below will mix into the oxygen minimum by the enhanced turbulence associated with the downslope front around day 186.7, or the upslope front on day 186.8. Likewise, the hypothetical enhanced nutrient layer is expected to mix with the waters above and below. The time-depth average turbulence values for the mooring site in Fig 4E are [<ε>] = 9±3×10^−7^ m^2^s^-3^, [<K_z_>] = 1.1±0.3×10^−2^ m^2^s^-1^, with [<N>] = 3.8±0.5×10^−3^ s^-1^. For the CTD-site, the time and [560 760] m average turbulence values are about half an order of magnitude lower, 2±0.7×10^−7^ m^2^s^-3^ and 4±1×10^−3^ m^2^s^-1^, respectively, with identical mean N. The turbulence dissipation rate is roughly the same at the two sites for the interior, and especially for z > -650 m (Fig 4D and 4E). The important difference is in the lower half of the sampled range. This difference is associated with the near-bottom approach of the warm water from above at day 186.7 and the following cool water from below at day 186.8, which are only found at the mooring site (Fig 4E). In that period, extending from the bottom upward, the CTD-site shows particularly low turbulence values (Fig 4D).

**Fig 5:**
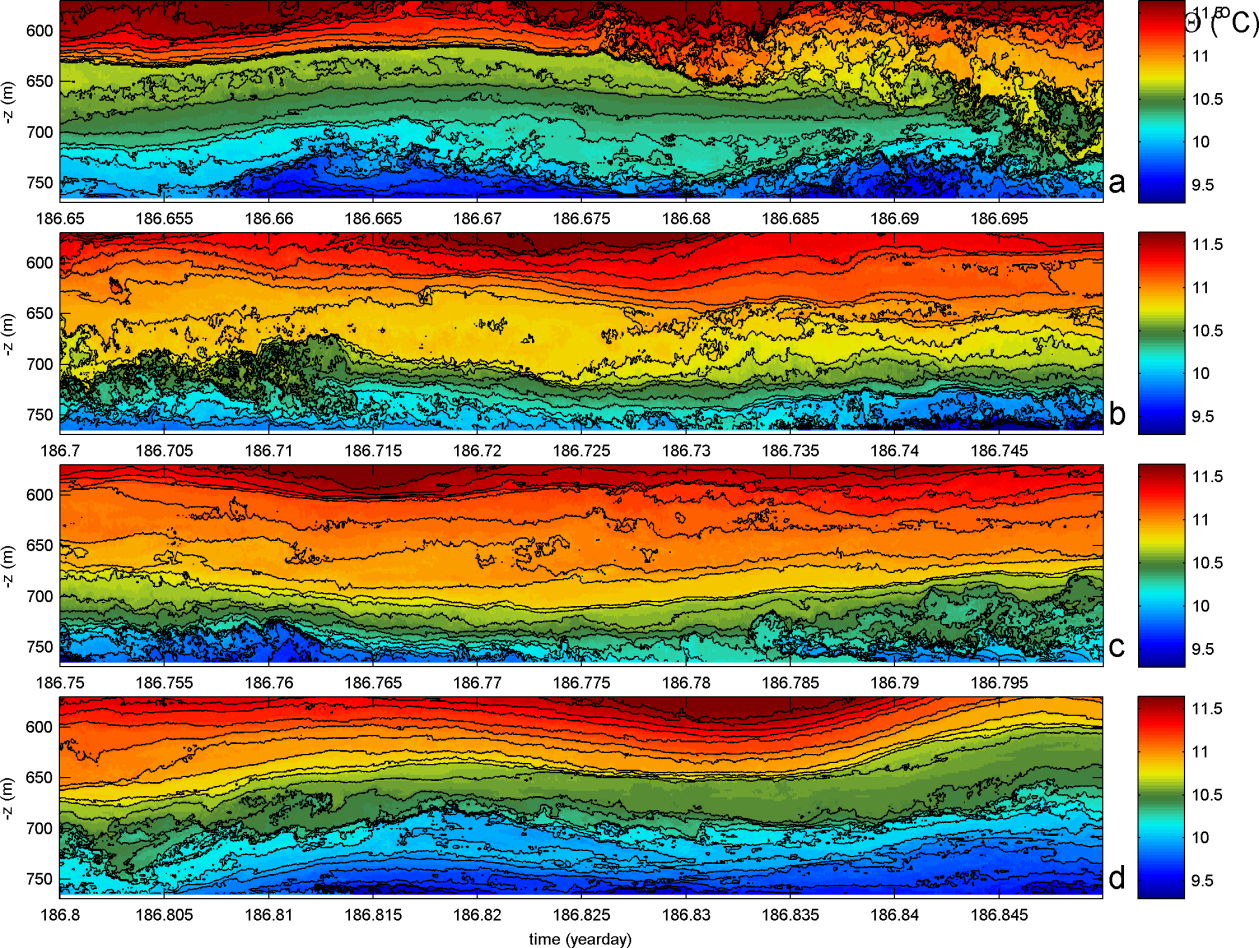
Details of time-depth series for moored T-sensor data. **Conservative temperature in four consecutive 1.25 hour periods of the center of Fig 4**. (a) Yeardays 186.65-.7. (b) 186.7-.75. (c) 186.75-.8. (d) 186.8-.85. Colour range the same for all panels, but slightly different from Fig 4A and 4B for better visibility of details. Black contours every 0.1°C.

Details of the turbulent waters at the mooring-site between days 186.65 and 186.85 are given in four consecutive panels of Fig 5. Initially, and typical for this seamount area, the pre-warming phase until day 186.675 shows overturning in two layers mainly, around 650 and around 730 m. From the contour-folding, both are seen to be dominated by shear-induced turbulence. The upper layer becomes disturbed first by the turbulence associated with warm water coming from above. It reaches near the bottom at day 186.7, in 50 m large overturns, but just not seems to touch the bottom. On day 186.735 some colder water moves up underneath, but most vigorously in terms of overturning after the warmest water reached the bottom, on day 186.78. The 50 m high turbulence associated with the cooler water moving up the slope does not appear as a very large front. The mixing trailing the front, however, reaches up to 100 m above the bottom in the entire panel Fig 5D. Above it, interior waters are relatively weakly turbulent, see also Fig 4D.

### Video transect (Fig 6)

The cross-ridge video transect shows the substrates are very heterogeneous, which provides many microhabitats for different species. The northwestern slope of the seamount is mostly covered with a blanket of biogenic carbonate sand, with the amount of calcareous coral rubble gradually increasing towards the summit. The southeastern slope of the seamount, the side of the mooring and the CTD-yoyo station, is mainly covered in coral rubble, interrupted between 720 and 770 m by fields of boulders. The shallowest part of the transect is characterized by a hard bottom which is occasionally covered by boulders or coral rubble.

**Fig 6:**
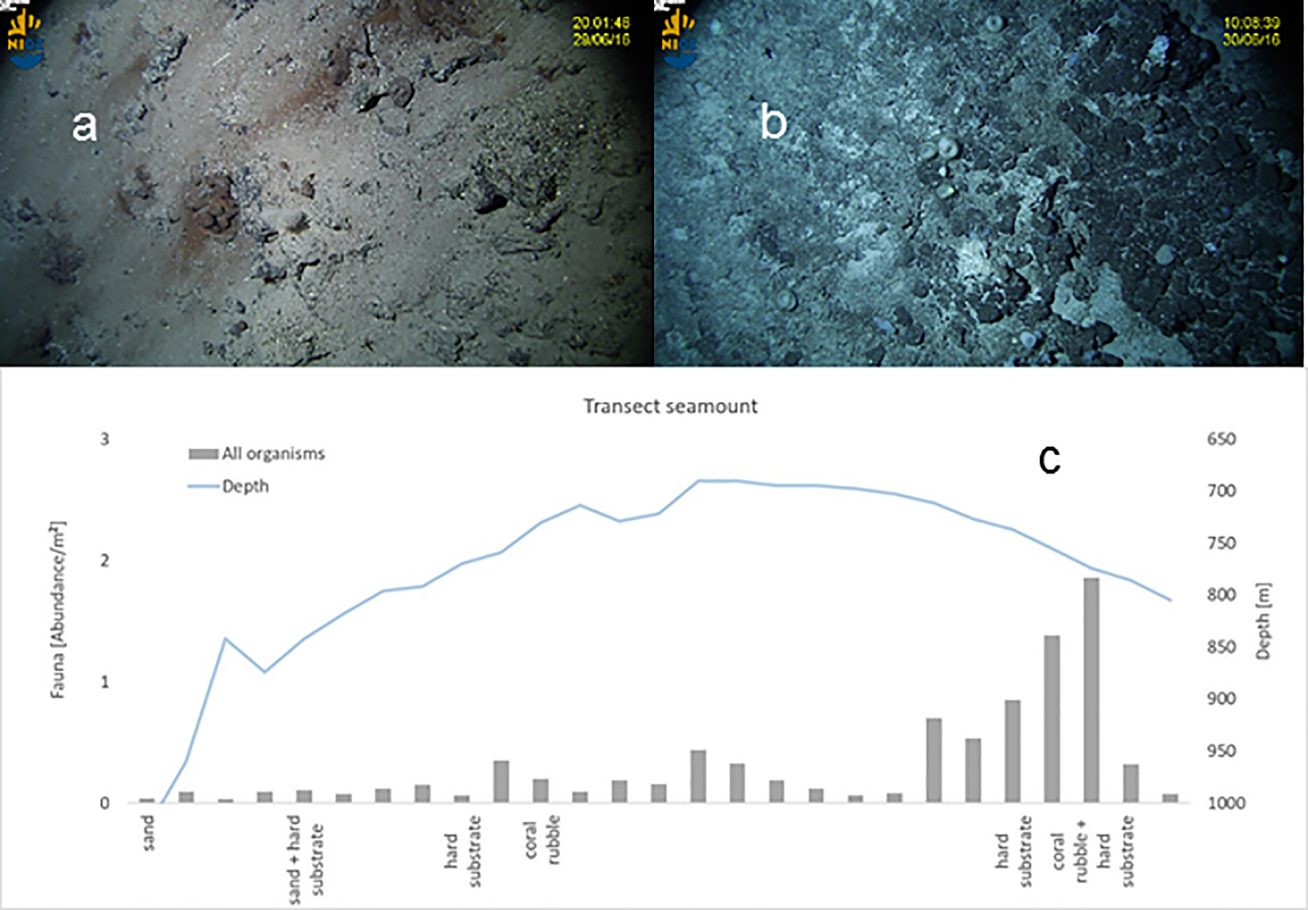
Video transect summary. (a) Representative picture from the northwestern slope, with sparse fauna and sandy bottom. (b) As a., but for coral rubble and boulders inhabited by sponges and gorgonians at the southeastern slope. (c) Abundance of all organisms, depth and substrata type along the transect.

At the southeastern slope, the region between 690 and 710 m was only scarcely inhabited. Slightly deeper, at 730 m, the highest abundance of organisms was found, whereas at a depth of >800 m low abundances were observed. We distinguished between 15 morphospecies of sponges plus 7 other non-sponge morphospecies. The highest density of sponges was observed at the southeastern slope of the seamount (0.3 ind/m^2^), whereas locally sponge abundances up to 1 ind/m^2^ were found (755 m). The sponges with the highest abundance and biomass in this area was *Pheronema carpenteri*. Gorgonians were restricted to the southeastern side and the summit of the seamount and were not found on the northwestern slope.

### Overview of moored observations

**Fig 7:**
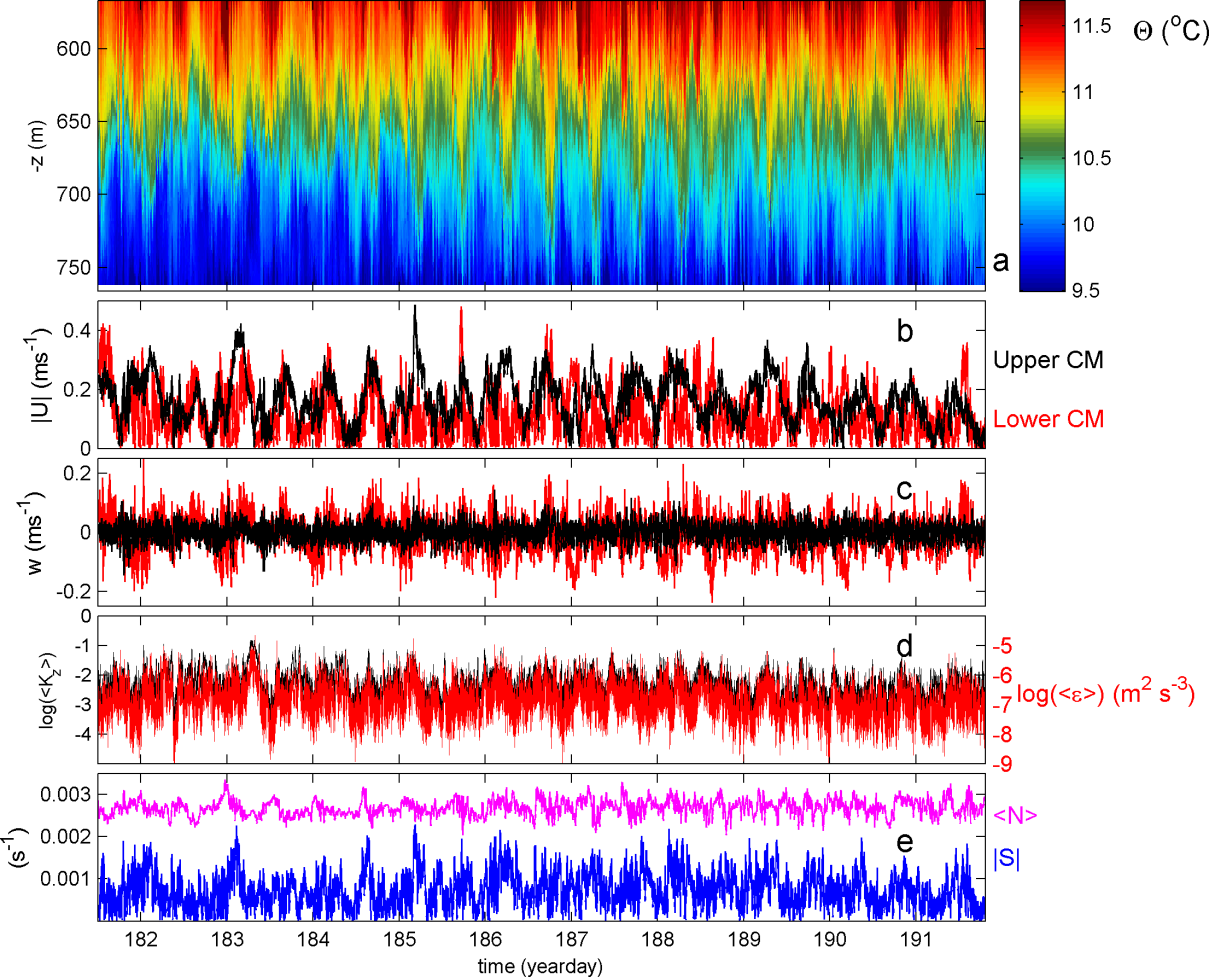
Time series overview of the moored data. (a) Conservative Temperature. (b) Current magnitude for upper (black) and lower (red) current meter. (c) Vertical current. (d) Logarithms of vertically averaged dissipation rate (red, scale to the right) and eddy diffusivity (black) from data in (a). (e) Vertically averaged buoyancy frequency (purple; from reordered profiles of raw data in (a)) and 200 m shear magnitude (blue, from data in (b)).

The one tidal period of Figs 4 and 5 is not exceptional for the seamount under investigation, although the measurements were made near springtide on day 186 (Fig 7). Even though the temperature variations have largest amplitudes around day 187 of >50 m crest-trough (Fig 7A), all other parameters barely show a spring-neap cycle. Other motions at frequencies higher than tidal occur intermittently having considerable amplitudes. For example, the current amplitudes (Fig 7B) show tidal bursts during neap tides (upper current meter CM, day 183.2) and shorter term bursts spiking above the tidal motions in both lower and upper CM on day 185. The near-circularly polarized tidal motions have a main axis direction perpendicular to (across) the crest. The 10-day averaged flow of 0.04 (bottom) and 0.1 m s^-1^ (top) is directed more or less southwest ward along the crest. The vertical current w does not show much spring-neap variation, neither smooth tidal variation (Fig 7C). It does show a spiky, burst-like variation, more or less occurring twice a day, both positive and negative or up and down, with additional, more frequent bursts. Note the 2–3 times larger amplitudes at the lower CM compared to the upper CM, with vertical currents reaching absolute values of 0.2 m s^-1^. The variations in w are reflected in the vertically averaged turbulence parameters (Fig 7D), which vary twice daily and at higher frequency. They vary over three (K_z_) and four (ε) orders of magnitude. The mean values for the entire 10 days period are similar to the ones for the tidal period in Fig 5, to within one standard deviation. A vague spring-neap cycle is visible in shear magnitude, computed between the two CMs nearly 200 m apart, with the exception of a peak on day 183 (Fig 7E). The 200 m mean buoyancy frequency from the reordered temperature profiles varies with smaller amplitudes than the variations in shear magnitude, with a mean about twice that of the shear. The vertical scales over which motions vary that drive the destabilizing shear larger than the stable stratification must therefore be considerably smaller than 200 m, more likely a vertical scale of 50 m or less. The latter vertical scale compares with the thickness of layers of turbulence activity observed in Fig 5.

**Fig 8:**
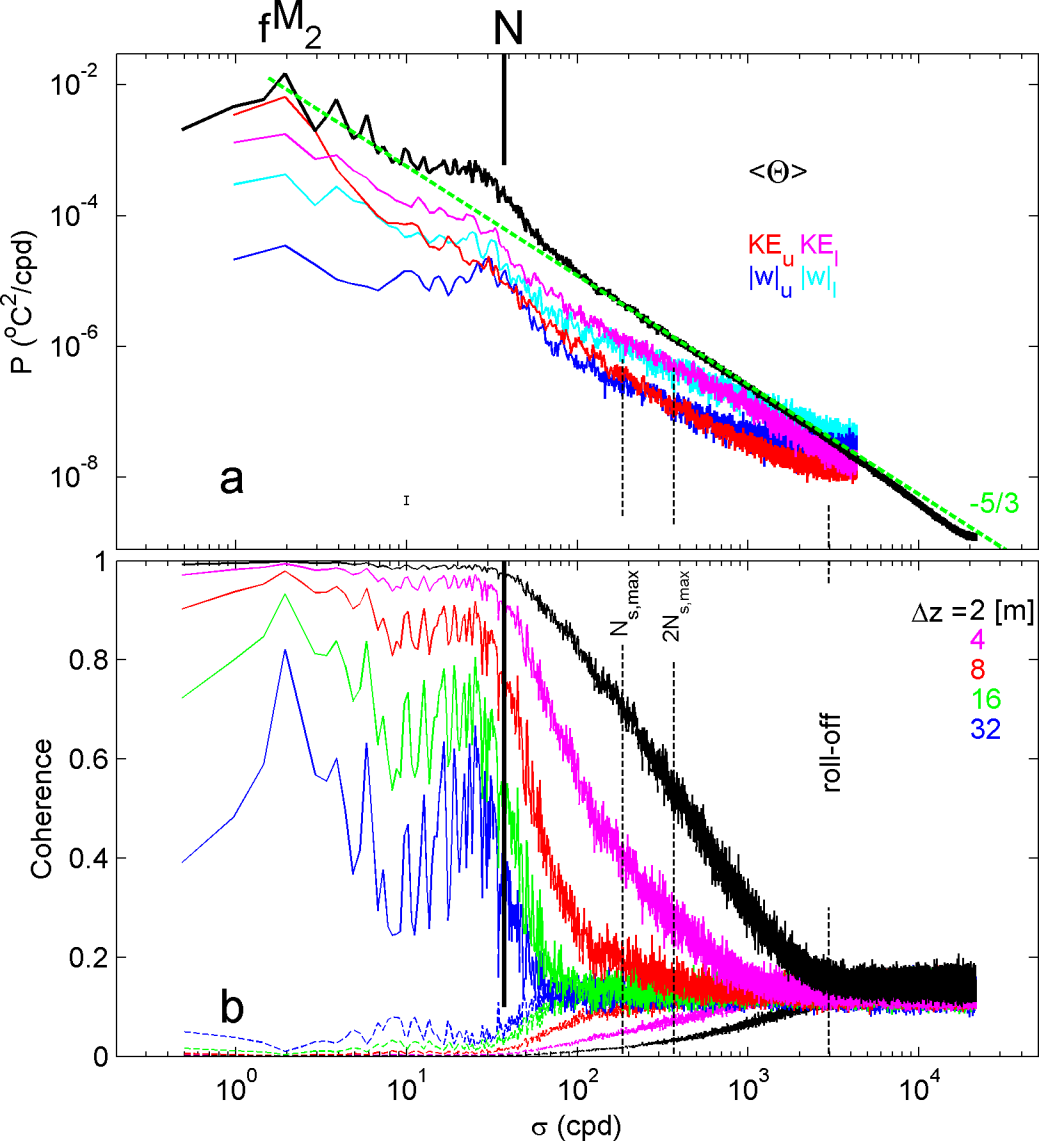
Spectral overview of moored observations. (a) Power spectra, weakly smoothed ~3 degrees of freedom ‘dof’ for CM-data (colours), and heavily smoothed ~1000 dof mean Conservative Temperature for all T-sensors. The dashed green line indicates the σ^-5/3^ slope for the inertial subrange. (b) Coherence level between all possible non-overlapping pairs of T-sensors, over vertical distances as given. The 95% confidence level is indicated by dashed lines of the same colour near the bottom (always coh lev < 0.2). The roll-off is where the coherence for the shortest vertical distance drops into noise. N_s,max_ indicates the maximum small-, 2 m, scale buoyancy frequency.

The short-term variability also exists in the temperature data, as is apparent in the overall variance spectra (Fig 8A). Because of the high number of sensors, the smooth Θ-spectrum shows semidiurnal tidal and higher harmonics peaks in the internal wave band [f, N], between inertial frequency f and the buoyancy frequency. Just before N, peaking at about 0.7N, the spectrum shows a considerable bulge, well-known in the open ocean [20], before dropping off into the turbulence inertial subrange within which the spectrum slopes with frequency σ at a rate of σ^-5/3^ e.g., [21]. Although the CM-spectra are much less smooth, because of single instrument data used in the computation, the vertical current also bulges near N, in particular for the upper CM. At, and just after, the near-N peak in w, the horizontal kinetic energy and the w-spectra have the same variance at the upper CM, and to within a factor of two the same variance for the lower CM. This reflects for motions at frequencies near N an aspect ratio near 1 for the upper CM and between 0.5 and 1 for the lower CM. The two CMs show more considerable differences between them. The KE-spectrum of the lower CM shows more variance at all frequencies except near the Nyquist frequency, which is noise dominated, and the semidiurnal (and lower) frequencies. This suggests a distinct redistribution from the internal tide, presumably the major internal wave source, to all other frequencies as turbulence increases towards the bottom. In contrast, the w-spectrum for the lower CM has more variance than that for the upper CM at all frequencies, with the two having the smallest difference in variance at N. In the internal wave band, the aspect ratio between horizontal and vertical motions is much larger at the upper CM, especially at semidiurnal frequencies, but with the exception near N earlier noted. It is relatively small at the lower CM, gradually becoming smaller with frequency.

In the Θ-spectrum, the inertial subrange stretches from approximately the maximum small-2 m-scale buoyancy frequency N_s,max_ to a roll-off frequency, here at about 80N. In both CM-spectra, the transition to the inertial subrange indicates the transition from E_k_ > w^2^, for σ < N_s,max_, to E_k_ < w^2^, for σ > N_s,max_. At these transition frequencies, which indicate the transition from 2D-anisotropic to 3D-isotropic full turbulence motions, the vertical temperature coherence is only significantly different from zero for the smallest vertical scales Δz ≤ 4 m (Fig 8B). In the transition, the Δz = 8 m coherence spectrum shows a little increase above the 95% significance level. Nevertheless, coherence is not very high, after being dropped steeply for σ > N. It is noted that at all scales investigated up to Δz = 32 m, coherence is significantly larger near N, compared to nearby frequencies higher ánd lower. Coherence near N has the same level as at the sixth-diurnal, third tidal harmonic.

## Discussion and conclusions

Turbulence values are high, half an order of magnitude higher at the mooring-site and about similar to within a factor of two at the CTD-site compared to values found elsewhere above sloping topography at about the same depths e.g., [17]. Such large values are (almost) comparable to those obtained in the near-bottom frictional zone of energetic shallow seas and tidal channels e.g., [22,23]. These values are 100–1000 times larger than observed in the open ocean, away from boundaries e.g., [24]. In contrast with the above sloping topography observations, here not only upslope moving cold-water fronts show large turbulence, but especially also interior motions, which, in general, do not reach the bottom. The turbulence is found in 20–50 m thick layers, and are likely shear-driven, as inferred from the overturning shapes. Over these vertical distances, internal wave motions near the buoyancy frequency are still as coherent as internal tidal harmonics. For higher frequencies, the vertical coherence rapidly drops for scales greater than 4 m. Only scales smaller than 4 m show significant vertical coherence well into the turbulence inertial subrange.

Between the maximum buoyancy frequency of these small scales and twice its value, the vertical current variance exceeds that of the horizontal currents. It does so not only in the generally more turbulent near-bottom waters, but also at 200 m from the bottom. At the latter distance, where turbulent motions are less energetic and internal waves smoother, the current meter also shows an aspect ratio of 1 near the larger-scale buoyancy frequency. The transition to full 3D-motions, with aspect ratio of 1, is also observed in a recent five-line T-sensor mooring in the lower 100 m above a sloping bottom [25]. Those measurements were however made in considerably deeper waters of 1740 m with half the stratification. The five-line mooring data notably showed a very different Θ-spectrum, with small quasi-coherent variations within the inertial subrange and without a smooth bulge near N.

The significance of the observed turbulence extent from the interior and from near the bottom for the local benthic fauna involve the supply of nutrients, oxygen and of particulate organic matter. High near-bed turbulence may promote resupension of organic particles sunken from the overlying water column. These particles could nourish filter-feeders such as sponges, crinoids and corals [26,27]. Also in our study bursts of near-bottom Reynolds stress and acoustic backscatter recorded by the near-bottom current meter showed a significant positive correlation (cross-correlation analysis, lag = 0, p < 0.05) indicating intermittent resuspension. Nutrients are essential for bacteria [28,29] which in turn are important for many benthic organisms either as food source or as symbionts. For instance, sponges may ingest bacteria while they also host a consortium of symbiotic bacteria [30,31]. Tropical corals are known to have symbionts and also ingest bacteria [32] whereas deep-sea gorgonians host a consortium of bacteria involved in nitrogen cycling e.g. [33]. Oxygen and nutrients generally display a negative relationship in the different watermasses of the oceans due to mineralization processes using oxygen thereby releasing nutrients [19]. The observations show a layer with depressed oxygen levels around 700 m at the CTD-site that becomes supplied by internal wave shear-induced turbulent mixing from above and below, every tidal cycle at least once. Such a depressed oxygen level layer is typical for the Atlantic Ocean, although most pronounced around the equator [8]. The depressed oxygen concentrations (170–180 μmol/kg), however, are not in a range that they immediately affect survival of for instance deep-sea corals [34]. Turbulent mixing of nutrients over (small) seamounts promoting locally enhanced primary production has been proposed as explanation for the rich fauna though retention time of the water might be too short for the organisms on the summit to benefit [3]. However, downstream, on the slopes, organic flux could give rise to enrichment of the benthic fauna.

In a study of the Condor seamount near the Azores island Faial, [6] found a similar belt of filter-feeding sponges on the slope as we did. As in our study, the belt was observed below the summit (720–860 m depth) of Condor seamount and dominated by the sponge *Pheronema carpenteri*. Hydrographic measurements on Condor seamount [35] revealed local maxima of enhanced mixing on the flank which could have a relation with the sponge belt. Mixing due to tidal currents interacting with sloping topography has in many places been linked to favorable conditions for filter-feeders. Along the slope of the Faroe-Shetland Channel internal bores cause resuspension leading to large number of bivalve filter-feeders at mid-depth [36,37]. Cold-water corals seem to thrive in places where tidal currents impinge on the slope such as the Faroer and Rockall Banks [38,39] while sponge belts in the Porcupine Seabight continental margin have been linked to breaking internal waves [40].

Following a 2D high-resolution numerical model of internal wave breaking above steep topography [41], we expect a horizontal extent of turbulence due to breaking waves of about 900 m. The present observations at the CTD-site are within 400 m from the bottom slope, and whilst being half an order of magnitude less turbulent than the ones estimated at the mooring-site, they still are O(100) times those found in the open ocean interior, e.g. in [23]. From the present observations we note that the rate and scale of turbulence overturning changes upward from about 70–120 m above the bottom. The largest difference between near-slope mooring-site observations and those obtained from the CTD-site are the near-bottom frontal turbulent motions, while the turbulent overturning in the layers away from the bottom show approximately the same turbulence levels.

Future studies should reveal whether our observations are portable to other seamounts. The coincidence of strong mixing and concentrations of filter-feeding corals and sponges in our and many other studies, calls for multidisciplinary studies encompassing hydrography, chemical oceanography and animal physiology. Such would form an important contribution for advancing predictive models of the distribution of key species and habitats e.g. [42] which thus far strongly depend on general aggregated data.
